# Prolonged On-Vine vs. Cold of *Actinidia eriantha*: Differences in Fruit Quality and Aroma Substances during Soft Ripening Stage

**DOI:** 10.3390/foods11182860

**Published:** 2022-09-15

**Authors:** Zhengxin Lv, Bin Ye, Xishi Li, Yanqun He, Qing Liu, Chunhui Huang, Dongfeng Jia, Xiaobiao Xu

**Affiliations:** 1 College of Agronomy/Kiwifruit Institute of Jiangxi Agricultural University, Nanchang 330045, China; 2 School of Agriculture and Biology, Shanghai Jiao Tong University, Shanghai 200240, China

**Keywords:** *Actinidia eriantha*, on-vine soft ripening, cold, fruit quality, aroma components, sensory evaluation, mathematical model

## Abstract

In order to find an efficient, economical and feasible method for soft ripening storage of kiwifruit, two softening methods (on-vine, cold) were utilized for the ‘Ganlv-2’ kiwifruit (*Actinidia. eriantha*) cultivar. A comprehensive evaluation was conducted on the quality changes in ‘Ganlv-2’ under different methods after fruit ripening by principal component analysis and mathematical modeling. Compared to kiwifruit under cold softening, kiwifruit treated with on-vine soft ripening had slightly greater sugar-acid ratios and flesh firmness and higher contents of dry matter, soluble solids, and soluble sugar. The titratable acid content was slightly lower in the on-vine group than in the cold group. The sensory evaluation results manifested little difference in fruit flavor between the two groups. However, at the end of the trial, the overripe taste of the on-vine group was lighter and the taste was sweeter than those of the cold group. More aromatic substances were emitted from the kiwifruit in the on-vine group. According to the mathematic model, there was no significant difference in fruit quality and flavor between the on-vine and traditional cold groups. The fruit in the on-vine group had a stronger flavor and lighter overripe flavor when they reached the edible state. This paper provided a novel storage method of *A. eriantha*, it can reduce the cost of traditional cold storage and reduce the pressure on centralized harvesting, and the feasibility of this method was verified from the fruit quality.

## 1. Introduction

As one of popular fruit crops in the world, Kiwifruit (*A. eriantha*) is a delicious perennial vine fruit crop of the Actinidia genus and has a unique flavor and delicious taste [1]. It is rich in ascorbic acid (AsA), dietary fiber, and a cultivar with mineral nutrients, which has several health benefits. The AsA content of Kiwifruit fruit is 3–4 times higher than that of A. chinensis [2]. Currently, excellent female cultivars and breeding lines, such as ‘White’ and ‘Ganmi-6’ [3,4], have been bred and selected, and the ornamental pollination of the superior male strain, ‘MG-15’ [5], has been bred. ‘Ganlv-2’ is a new cultivar of high-quality kiwifruit that was recently selected and bred by our research group. It has green flesh and a sweet flavor that is fresh, fragrant, and strong. It is selected and bred by natural variations in the wild *A. eriantha* population.

However, due to its short maturity period and insufficient attention and investment in post-harvest processing in China, the storage technology is relatively backward, and the fresh fruit become highly perishable [6]. Cold storage is one of the most widely used preservation techniques in commerce today; it is used in almost all fruit crops, especially those which can tolerate cold [7,8,9,10,11]. On-vine soft ripening is a delayed postharvest technique that has been applied for the storage of mango and grapefruit [12,13]. This method can extend the harvest period and it also can maintain good fruit quality of other fruit crops. In commercial trade, kiwifruit tend to be harvested at a mature but unripe state; a previous study about climacteric fruit has reported that fruit are picked when their soluble solid content reaches ~7.0% [14]. Based on many years of field experiments, *A. eriantha* in the harvest period is not likely to have fruit fall off the vine, and it can maintain good fruit quality for a long time. There are two main reasons for this phenomenon. The first possibility is that compared with the fruit of *A. chinensis* and *A. deliciosa*, the fruit of *A. eriantha* is smaller, which makes it more difficult for it to fall off during the soft ripening process. The second possibility is that the content of AsA in *A. eriantha* is very high. AsA is an antioxidant and enhances the activities of various antioxidant enzymes; more H_2_O_2_ is removed, and this may delay the formation of the abscission layer between fruit and carpopodium, which makes the fruit fall off naturally [15,16]. Thus, there will be a long wait before the fruit ripen and reach the edible state. The storage time of different fruit species varies greatly. For example, some citrus cultivars can reach a storage time of 90 d [17]. Burdon et al. studied the impacts of on-vine soft ripening and cold storage on the softening rate of ‘Hayward’ kiwifruit; in storage, the initial slow phase of softening was seen only in less mature fruit harvested before any increase in softening rate had occurred on the vine. Maturing ‘Hayward’ fruit developed a capacity to soften in response to low temperatures 1–2 weeks before the on-vine period of faster softening commenced [18]. Aroma substances are a key factor that affects the flavor quality of fruit [19]. Sour and sweet taste and aroma constitute the flavor quality of fruit [20]. Aroma is produced as fruit mature and changes during postharvest storage [21,22]. Prior research has reported the volatile aroma substances of many kiwifruit cultivars, but less so on *A. eriantha* [23].

Mathematical models such as linear equations, one-dimensional polynomial equations and logistic equations are often used to map the dynamics of fruit growth and development. In production, Da S and Frédéric B preferred to analyze the relationship between fruit growth indices and development time, and to derive the equation to analyze the relationship between growth rate and development time of each index by polynomial regression [24,25]. Yuan et al. fitted the growth and development dynamics of kiwifruit fruit well by linear equations [26]. A new shape equation for Hayward kiwifruit has been developed by Olatunji et al., which can predict the fruit shape of kiwifruit within 4% error [27]. A model of kiwifruit berry development was presented, building on the model of Fishman and Génard used for peach fruit; the resulting model showed close simulation of field observations of fresh weight, dry matter, starch, and soluble solids in kiwifruit [28]. Mathematical models were extensively used for other fruit trees such as apples, grapes, bananas and pears [29,30,31,32,33]. The key inflection point of fruit growth can be determined by constructing a fruit growth model, which can provide a reliable basis for high yield and quality production of fruit trees [34]. The soft ripening of climacteric fruits changes with time and space; the law of indices is closely related to fruit quality and preservation period [35]. In the theoretical study of fruit growth and development, the model of fruit growth dynamics can be used as an evaluation and prediction method for fruit growth and yield [36]. In this paper, we use a similar method to analyze the difference between two soft ripening methods of ‘Ganlv-2’ kiwifruit.

On-vine soft ripening can reduce the cost and postharvest losses and improve the economic benefits of sightseeing and picking. In this paper, we explore changes in fruit quality of *A. eriantha* under on-vine soft ripening, ‘Ganlv-2’ was selected as the test material (Fengxin county, Jiangxi Province). Hence the objective of this study was to uncover the changes of the main fruit qualities and aroma substances during softening of two soft ripening methods, and the feasibility of on-vine soft ripening was analyzed. These findings are discussed in the context of potential commercial impacts from a better understanding of the effect of different storage methods on kiwifruit softening.

## 2. Materials and Methods

### 2.1. Experimental Materials

**Figure 1:**
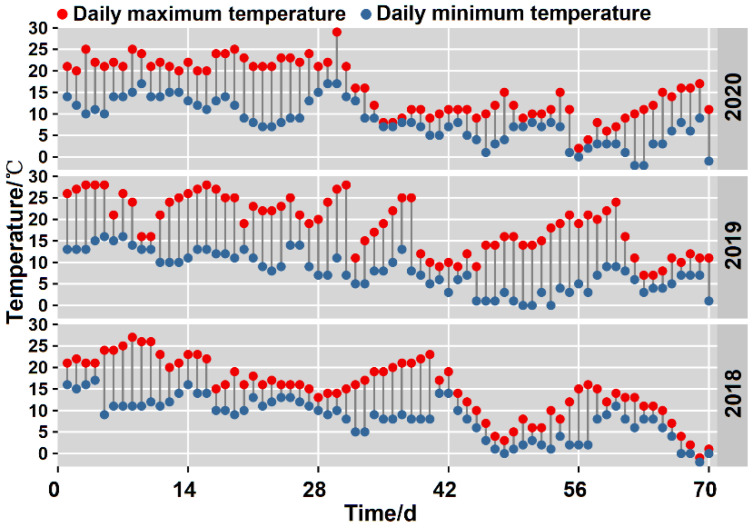
The daily mean temperature of sampling period in Fengxin County.

The experiment was conducted from 2018–2020, the repetition effect between years was good and the differences were small; therefore, this study selected the data of 2019 for analysis. The sampling start date of 2019 was October 16, samples were collected when the soluble solid content of fruit reached approximately 7.0% (physiological maturing stage), and sampling ended when the fruit appeared to be significantly wrinkled or if a large number of the fruit dropped from the vines [37]. Vines were 5 years old and normally managed, and the growth rates were relatively consistent. ‘Ganlv-2’ is a new high-quality kiwifruit cultivar selected from wild *A. eriantha*. The mother plant came from Magushan in Jiangxi Province and was domesticated in the sampling site of this study. A horizontal large trellis was utilized for cultivation, with a line space of 3 m × 4 m. The rootstock was *A. deliciosa*, and the ratio of male and female plants was 1:8. Fruit with uniform size, no structural damage, no disease, and no pests were selected as experimental materials for on-vine soft ripening. Ten vines were selected for each method. For fruit in the on-vine group, they were not harvested but were rather left hanging on the vine. Appropriate protective measures were taken, and the fruit were ripened under natural conditions. Figure 1 shows the daily mean temperature of the sampling site during the sampling period of 3 years that ranged from 11.18–12.46 °C. A total of 400 fruit were picked from the orchard under the same conditions and stored at a low temperature after the removal of field heat for 24 h with the temperature set to 0.5 °C. Starting from 0 d of cold softening, 50 fruit were obtained from the vine every 14 d and transported to the laboratory to determine fruit quality alongside fruit in the cold softening group. Sample collection and measurement were carried out for 3 biological replicates.

### 2.2. Fruit Quality

Soluble solids were determined using a hand-held explicit sugar meter PAL-1 (ATAGO Co., Ltd., Tokyo, Japan). Eight fruit were randomly selected from each group. After the fruit were transported to the laboratory, they were peeled, cut across the diameter into 3–5 g slices, and dried at 65 °C to a constant weight. The dry matter content was calculated as follows [38]:

Dry matter (%) = fruit dry weight (g)/fruit fresh weight (g) × 100(1)

Calculations were repeated 5 times for each method.

AsA was measured by the 2,6-dichlorophenol indophenol titration method using three replicates [39], and fruit firmness was determined by randomly selecting 5 fruit from each group. Fruit were washed and the skin was dried, followed by the removal of the pericarp. The firmness was measured at the same site in each fruit. A texture analyzer SMSTA-XTPlus (Stable Micro System Co., Ltd., Surrey, UK) was employed to assess fruit firmness with the following parameters: probe size, 2 mm (diameter) × 24.5 mm (length); drilling, 6 mm; test rate, 1 mm/s; test mode, TPA. The fruit were rotated 90° after every measurement and 5 fruit were measured. Based on the characteristic curve of puncture firmness, the parameters of flesh firmness and cohesiveness were calculated [40]. The soluble sugar content was detected using the improved anthrone colorimetric method [41]. The method of titratable acid referred to the report of Ma et al. [42].

Aroma substances were determined by weighing 6 g kiwifruit flesh tissues from frozen samples and grinding them into a powder with liquid nitrogen. The samples were placed in a 20 mL headspace bottle. Then, 5 mL saturated sodium chloride solution was added, and the bottle was sealed. After equilibrating in water at 40 °C for 10 min, 65 μm PDMS/DVB (Supelco Co., Ltd., Bellefonte, PA, USA) was extracted with an extraction needle at 250 °C for 30 min. Then, the extraction needle was inserted into an injection port of an Agilent 7890A-5975 gas mass spectrometer (Agilent Inc., Santa Clara, CA, USA) for 5 min. The chromatographic column was a DB-WAX capillary column (30 m × 0.25 mm × 0.25 μm) (Agilent Inc., Santa Clara, CA, USA). The condition of the gas chromatography-mass spectrometry (GC-MS) gas phase and mass spectrometry were set as previously reported by Zhang: the spectra of unknown compounds were searched using a computer and matched with the NIST14 mass spectrometry database to determine the chemical constituents of volatile substances (material characterization); Normal-alkane of C7–C40 was determined and analyzed in the same way as the sample, and the Kovats Index (KI) of compounds was calculated according to the results of n-alkanes and compared with the values in the reference. KI was calculated in the equation; under the same GC-MS procedure, KI was compared with the retention time of the standard materials. The relative content of each component was acquired based on the peak area normalization method (quantitative method) [43,44].

(2) $$KI(x)=100\times n+\frac{\mathrm{RT}(x)-\mathrm{RT}(n)}{\mathrm{RT}(n+1)-\mathrm{RT}(n)}\times 100$$

In the equation, KI (*x*) represents the Kováts retention index of substance *x*, RT (*x*) represents Kováts retention time of substance *x*, and RT (n) and RT (n + 1) represents retention time of n and n + 1 alkane carbon atoms.

### 2.3. Sensory Evaluation

Table 1 based on observation and evaluation of the odor, color, texture and other indicators. The score is a 10 point system (sensory evaluators may not communicate with each other in the evaluation process, otherwise the judgment of the results will be affected and cause errors). After removing the lowest score and the highest score, the average value is taken for the results. For the sensory evaluation, ten kiwifruit were selected from each method, with one for each team member. After evaluating one sample, the team members rinsed their mouths and rested for 15 min before the evaluation of the next sample. Sensory evaluation was performed immediately after each sampling [45,46].

### 2.4. Data Analysis

The experimental data were statistically processed using Microsoft Excel 2010 software and analyzed by SPSS v24.0. SPSS v24.0 was used for PCA, modeling and best-fit analysis, and Pearson’s method was used for linear and partial correlation analysis. Taking harvest days as x and fruit growth and quality indicators as y, the optimal model was determined by linear equations, one-dimensional polynomial equations and logistic equations fitting analysis. The Z-core standardization method was adopted to non-dimensionally process the original data. he raw data of all measured indicators was imported into SPSS for analysis. All data were used as variables, and correlation analysis was undertaken. The extraction factor was based on the eigenvalue. After outputting the results, the results of kmo and Bartlett’s test were verified to determine whether the data were suitable for principal component analysis. Fit analysis was performed by Origin Pro 2021, testing with multiple fitting methods, screened and judged by the value of R^2^. Finally, it was considered that the multiple regression equation was more suitable for the data of this research. Analysis of variance (ANOVA) and Duncan’s multiple analysis were carried out to determine significant (*p* = 0.05) differences between the two groups. Trends and figures within the data were described graphically using Origin 2021 pro and R Studio.

## 3. Results

### 3.1. Differences in Fruit Quality

**Figure 2:**
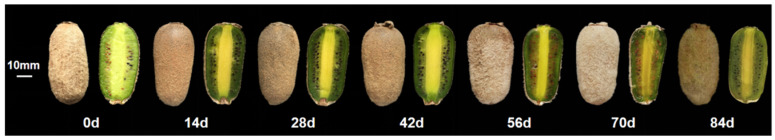
Dynamic changes of on-vine soft ripening fruit.

The flesh was light green on the 0 d and turned to dark green on the 14 d, with the core beginning to become yellow on the 70 d, which was consistent with the sensory evaluation results. Most of the fruit collected on the 84 d had different degrees of water loss and exocarp shrinkage, with severe flesh water loss and overripe taste. The poor flavor of the fruit on the 84 d almost lost its edible value. Therefore, the sample time was up to 70 d (Figure 2).

**Figure 3:**
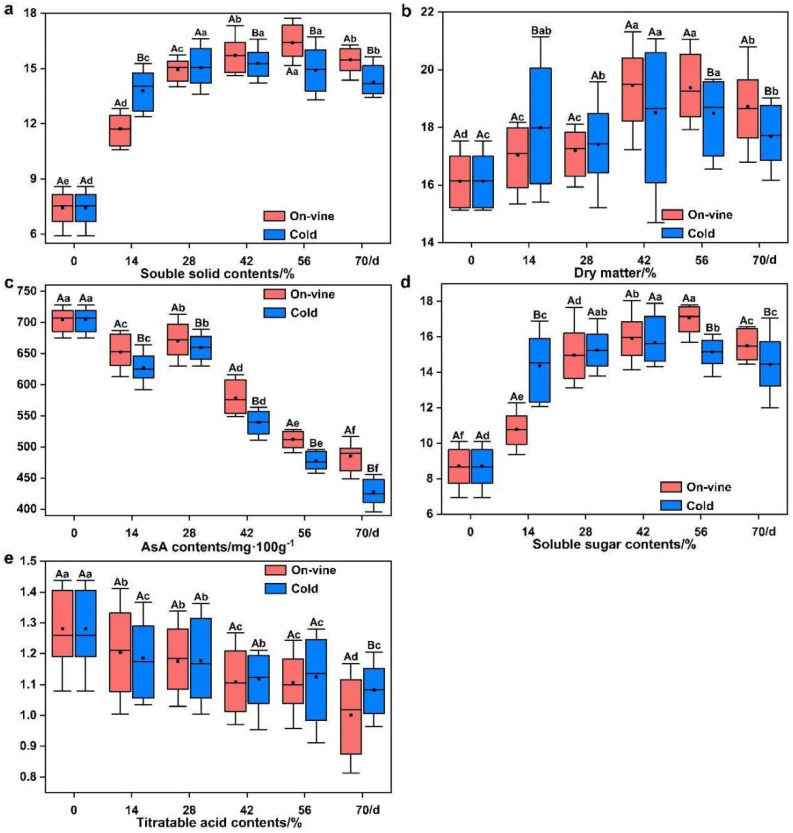
Changes in fruit quality with different softening methods. (**a**) Soluble solid contents. (**b**) Dry matter. (**c**) AsA contents. (**d**) Soluble sugar contents. (**e**) Titratable acid contents. Data are expressed as mean ± standard deviation of three biological repetitions. Error bars represent standard deviations of means. Different capital letters represent the difference between the two softening methods at the same harvest time, which are significantly different at *p* < 0.05. Different lowercase letters represent the difference in the different harvest times using the same method, which were significantly different at *p* < 0.05. Data were analyzed with Duncan’s multiple analysis.

Soluble solid content is one factor applied for judging fruit maturity. Changes in soluble solid content of the cold group were similar to those in the on-vine group (Figure 3a). At 14 d, the soluble solid content in both groups increased rapidly and then slowed down. The on-vine group and the cold group showed a slight decrease in soluble solid content at 56 d and 42 d, respectively. The soluble solid content of the on-vine group peaked at 16.33% at 56 d, and that of the cold group peaked at 15.32% at 42 d. The soluble solid content of the on-vine group and the cold group were 15.63% and 14.32% respectively at 70 d.

Dry matter is an essential index of the accumulation of organic compounds and the composition of nutrients in fruit. The dry matter content elevates as fruit mature. The change in the dry matter content of the cold softening group was similar to that of the on-vine group. Both groups exhibited volatile fluctuations in dry matter content. However, the range of the changes in both groups was not large (Figure 3b). The dry matter content of the on-vine group peaked at 19.52% at 42 d, and that of the cold group peaked at 18.61% at 56 d.

The AsA content of ‘Ganlv-2’ was between 425.70 and 701.39 mg·100 g^−1^ (Figure 3c). During the sampling period, similar trends of the AsA content existed in both groups. Over time, as the fruit matured in both groups, the AsA content enhanced slightly after 14 d and reduced slightly after 28 d. Moreover, the fruit in both groups continued to demonstrate a decline in the AsA content after 28 d. The AsA content of the on-vine group was slightly higher than that of the cold group, with no statistically significant difference. By the end of the experiment, the AsA content was 482.30 and 425.70 mg·100 g^−1^ in the on-vine group and the cold group, respectively.

The soluble sugar content augmented as organic compounds accumulated due to postharvest respiration. The soluble sugar content of the on-vine group peaked at 16.89% at 56 d, and that of the cold group peaked at 15.53% at 42 d (Figure 3d). The soluble sugar content of the on-vine group began to diminish slowly after 56 d, and that of the cold group began to decrease slowly after 42 d. The soluble sugar content was higher in the on-vine group than in the cold group. However, the gap was gradually narrowed over time. The final soluble sugar content of the two groups was 15.68% and 14.40%, respectively.

The changes of the titratable acid content exhibited a fluctuating trend during the soft-ripening process. Eventually, the titratable acid content of the two groups was reduced slightly. At 70 d, the titratable acid content of the on-vine group and the cold group were 1.00% and 1.09%, respectively (Figure 3e).

**Figure 4:**
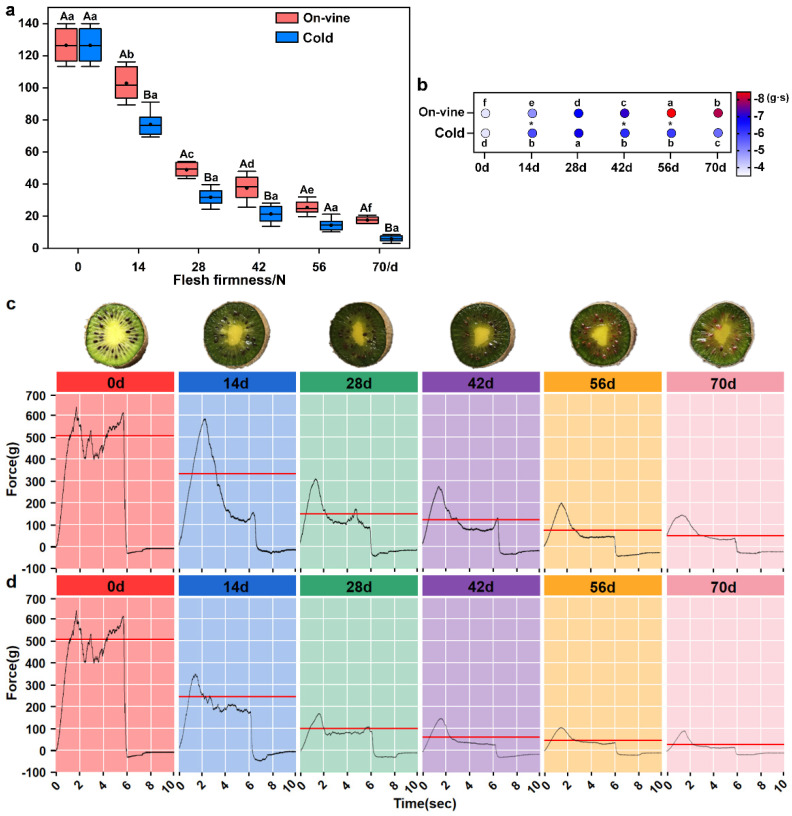
Changes of flesh firmness and cohesiveness. (**a**) Flesh firmness. (**b**) Flesh cohesiveness. (**c**) The TPA characteristic curve of the on-vine group. (**d**) The TPA characteristic curve of the cold group. Data are expressed as mean ± standard deviation of three biological repetitions. Error bars represent standard deviations of means. Different capital letters or ‘*’ represent the difference between the two softening methods at the same harvest time. Different lowercase letters are significantly different at *p* < 0.05. Data were analyzed with one-way analysis of variance (ANOVA) and Duncan’s multiple range tests. The red line in (**c**,**d**) represents the calculated flesh firmness, and this value was the average of all biological and technical repetitions of different methods in each collection period. Only one characteristic curve was selected to represent the test result, and other characteristic curves had only slight errors.

Flesh firmness is a key trait utilized for the identification of fruit quality. Fruit that are too hard or too soft are generally not ideal for consumption. The flesh firmness of kiwifruit under different softening methods declined considerably in the post-ripening stage (Figure 4a). The trends of flesh firmness changes were almost the same for both groups—fast initially and slow afterward. The firmness of fruit from the on-vine group was slightly greater than that of the cold group. At 70 d, the fruit firmness of the on-vine and cold groups was 17.54 and 6.08 N, respectively, indicating the similar freshness of fruit. The variation tendencies of flesh cohesiveness were similar for both groups, and diminished after reaching the peak value. During the whole softening period, the change range of the on-vine group was 220.10% (−3.83–−8.43 g·s), and that of the cold group was 180.79% (−3.83–−6.93 g·s). However, the on-vine group had higher cohesiveness than the cold group, and the range of variation enhanced after 42 d (Figure 4b). As manifested by the TPA characteristic curve (Figure 4c,d), the softening rate of peel and flesh of fruit in the on-vine group was lower than those in the cold group with harder flesh texture, which delayed the senescence of the fruit to a certain extent. There was an insignificant difference in the flesh cohesiveness between the two groups.

### 3.2. Differences of Aroma Components

**Figure 5:**
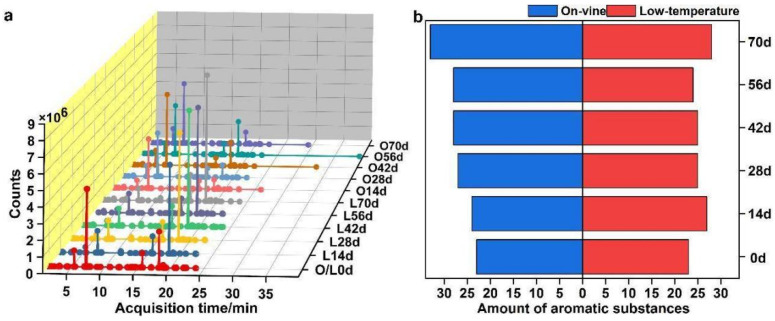
Quantitative analysis of aroma compounds. (**a**) Total ion current flow diagrammatic sketch of aroma substances. (**b**) Quantity of aroma substances under different softening methods in each period.

Figure 5a manifests the total ion flow in all periods. A total of 48 aroma components were detected in 7 categories, and there were 23–33 aroma components during the period of each softening method. The total relative content of each period was effective >95%. In the comprehensive comparison, the aroma species and relative content were higher in the on-vine group than in the cold group (Figure 5b).

**Figure 6:**
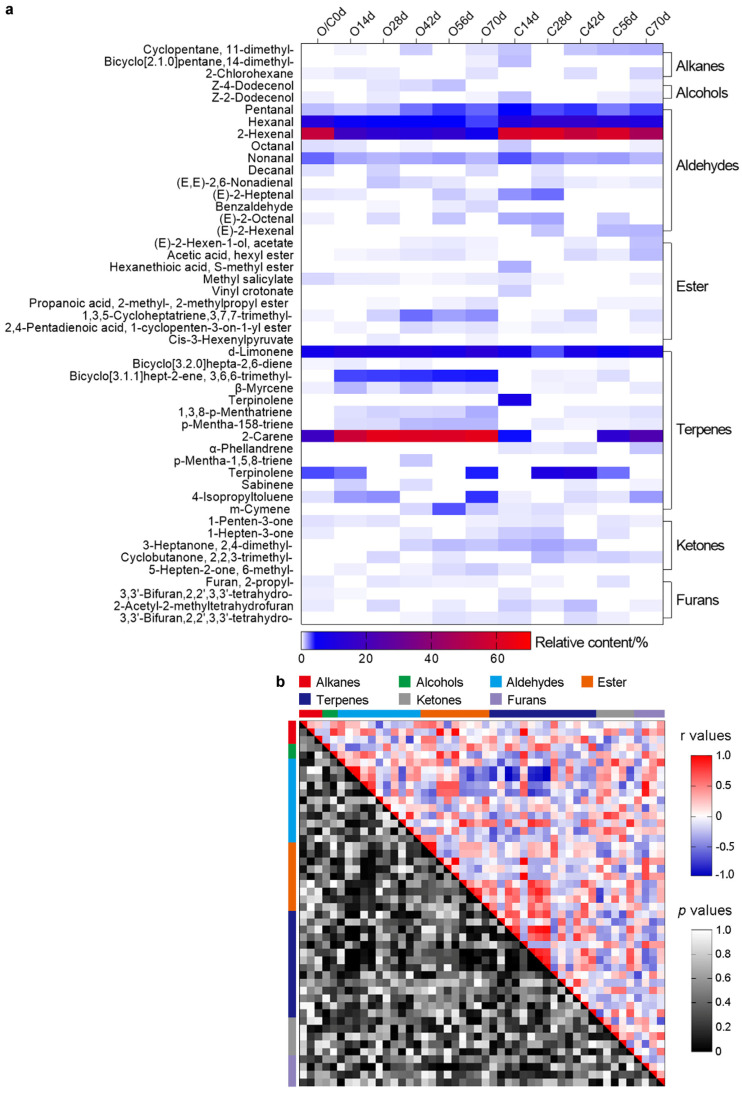
Identification and analysis of aroma components. (**a**) The relative content of aroma components in ‘Ganlv-2’ of different softening methods. (**b**) Aroma compounds correlation/significance of on-vine and cold group. In the colored area, rectangles represent r values resulting from Pearson correlation coefficient computation (see correlation color key). In the black and white area, rectangles represent *p* values respective to Pearson correlation coefficient (see Significance color key). Z−score transformation was employed to enable correlation computation. Note: ‘O’: on-vine group; ‘C’: cold group.

The basic aroma components detected in kiwifruit under different softening methods are listed in Figure 6a. The substances that were found in different periods under different softening conditions included pentanal (0.39–1.98%), hexanal (1.48–15.19%), 2-hexenal (6.61–61.44%), d-limonene (1.34–13.11%), and nonanal (0.56–1.37%). A total of 3 alkanes were detected under both softening conditions, but their contents were low with <1% of the total amount. Only 2 alcohols were found, and their contents were also low. However, the expression of these 2 alcohols was higher in the on-vine group than in the cold group. A total of 11 aldehydes were detected, and their contents significantly and continuously decreased. The contents of aldehydes in the cold group decreased slowly after reaching a peak of 81.44% after 28 d, which was the highest value recorded in the entire experiment. Among these, 2-hexenal showed the highest content. The species and content of esters increased as the fruit ripened. The species and the overall content of esters were more in the on-vine group than in the cold group. The alkene and terpene contents were high in both groups. In the on-vine group, these contents elevated substantially as the fruit matured and reached their highest values after 14 d. The peak value of terpenes reached 83.26% after 70 d. The substance with the highest content was 2-carene. The total content in the cold group exhibited a decreasing and increasing trend, but not to a large extent. A total of 5 ketones were detected, and their contents in the on-vine group enhanced continuously and reached the peak value of 1.15% after 70 d. However, their contents in the cold group firstly increased and then reduced, with a peak of 1.92% after 28 d. A total of 4 furans were found. Their contents were all low at the beginning and diminished at the end of the experiment. There were some differences in the types and contents of numerous aroma substances between both groups, and unique aroma substances were produced at 70 d.

To observe the relationship between aroma components during fruit soft ripening, we analyzed the correlation between them (Figure 6b). The average correlation coefficient of each aroma component was 0.31, the positive correlation coefficient accounted for 48.14%. Three groups had a correlation coefficient of 1: vinyl crotonate and hexanethioic acid, s-methyl ester; terpinolene and hexanethioic acid, s-methyl ester; vinyl crotonate and 1,3,8-p-Menthatriene.

### 3.3. The Contents and Variations of Various Aroma Components

**Figure 7:**
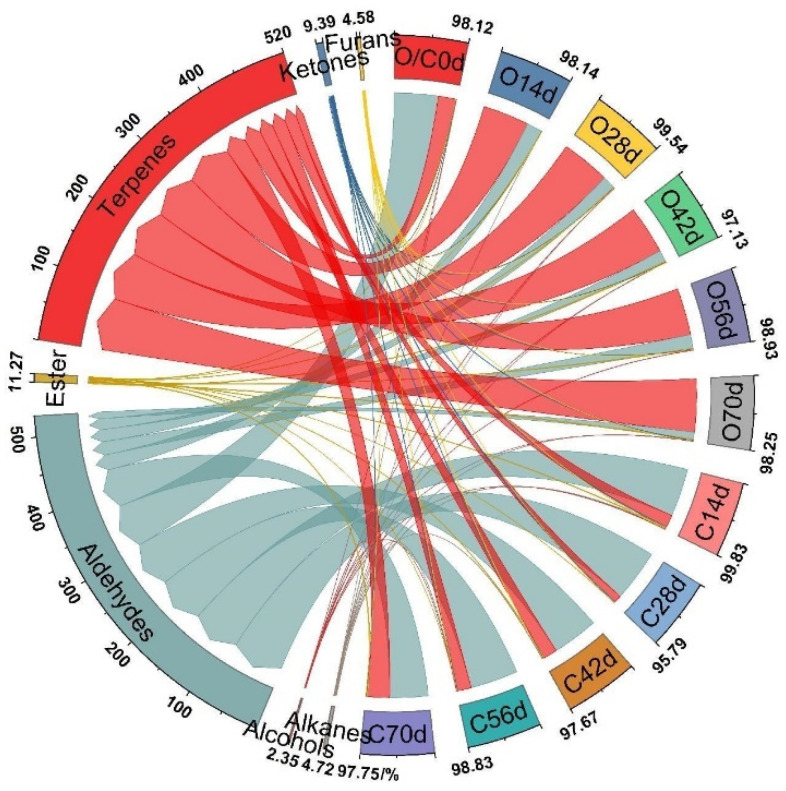
The relative contents of different types of aroma substances in the fruit under two method conditions. The groups represented by outside—inside in the ring graph were O/C0, O14, O28, O42, O56, O70, C14, C28, C42, C56, C70 d, respectively.

The changes in the relative contents of each kind of aroma component in different periods of *A. eriantha* under different softening methods are demonstrated in Figure 7. A total of 3 kinds of alkanes were detected after the two methods, with low contents (<1% of total amount). A total of 2 kinds of alcohols were detected, with low contents (<0.5%). A total of 11 kinds of aldehydes were detected. The relative contents of aldehydes were conspicuously decreased in the on-vine group with the extension of softening period (69.22% (0 d) to 10.61% (70 d)). It reached the peak value of 81.44% in the cold group on the 28 d and began to reduce slowly, while still high on the 70 d (60.65%); this was the highest content in the cold group during the whole softening period. Among these aldehydes, the content of 2-hexanal was the highest, ranging from 46.97% to 61.44%. A total of 9 kinds of esters were found, with low total content (0.24–1.61% in the cold group and 0.45–1.85% in the on-vine group). A total of 14 kinds of terpenoids (the second-largest type of aroma compounds in the two groups) were detected. The contents of terpenoids notably augmented in the on-vine group with the extension of the softening period, reaching the highest content of aroma compounds from 14 d and reaching the peak of 83.26% on the 70 d. The relative contents of terpenoids were 11.75–33.64% in the cold group. A total of 5 kinds of ketones were detected. The contents of ketones were continuously increased in the on-vine group, reaching the peak value of 1.15% on the 70 d. The peak value of ketone contents in the cold group was 1.92% on the 28 d. A total of 4 kinds of furans were found, which exhibited low contents, ranging from 0.14% to 0.83%, and the contents reduced on the 70 d. It was noted that there existed differences in the types and contents of aroma compounds between the two groups. Aldehydes were dominant in the picking period in the two groups. The contents of terpenes in the on-vine group gradually became the highest with the extension of softening period, while the aldehydes were always predominated in the cold group.

### 3.4. Variations of Main Aroma Components

**Figure 8:**
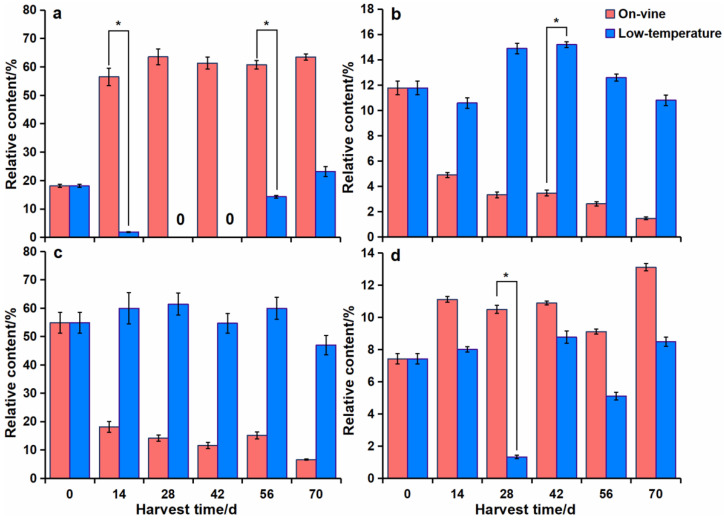
Changes of main aroma components of kiwifruit under the two softening modes. (**a**) 2-carene. (**b**) Hexanal. (**c**) 2-hexenal. (**d**) d-limonene. Error bars represented standard deviations of means. ‘*’ represented significant difference at *p* < 0.05.

2-hexanal, hexanal, 2-carene, and d-limonene were the four aroma compounds with the highest relative content under the two methods. 2-carene was the aroma compound with the highest relative content in the on-vine group, which elevated noticeably from the 14 d, reaching 63.55% (the peak value) on the 28 d and 63.44% on the 70 d. Additionally, 2-carene was the second-largest aroma component in the cold group at the end period (Figure 8a). The content of hexanal diminished with time, being 11.77% on the 0 d and 1.48% on the 70 d. The content of hexanal showed a ‘downward-upward-downward’ trend in the cold group, and its peak appeared on the 42 d (15.19%) and dropped to 10.80% on the 70 d (Figure 8b). 2-hexanal was the aroma compound with the highest content in the cold group, reaching 61.44% (the peak value) on the 28 d and then slightly decreasing to 46.97% relative content on the 70 d. The content of 2-hexanal reduced as the softening period prolonged in the on-vine group, with 54.88% relative content on the 0 d, which was then considerably diminished to 18.12% on the 14 d and 6.61% on the 70 d (Figure 8c). The content of d-limonene in the on-vine group did not change much, and its peak value appeared on the 70 d (13.11%). The content of d-limonene was lowest on the 28 d (1.34%) in the cold group, and then fluctuated and increased to 8.49% on the 70 d (Figure 8d). The aroma descriptions of 2-hexanal, hexanal, 2-carene, and d-limonene were herbaceous flavor, orange flavor, sweet fragrance, lemon flavor and herbaceous flavor, respectively (Table 2).

### 3.5. Effects of Different Softening Methods on the Sensory Quality of ‘Ganlv-2’

**Figure 9:**
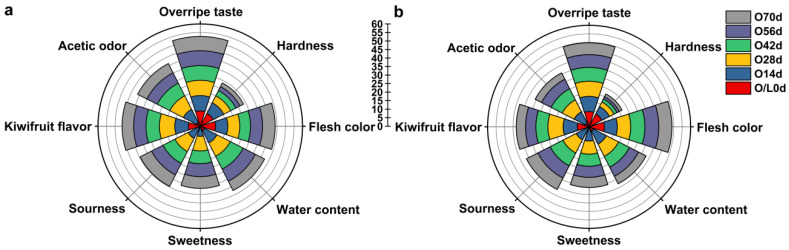
Results of the sensory evaluation. (**a**) On-vine group. (**b**) Cold group. The final score is the sum of the average scores of all organoleptic properties.

The sensory quality of the cold group at different periods was evaluated, scored, and ranked. The scores of both groups at each period were summarized (Figure 9). An insignificant difference in the sensory index was observed between the two groups, and the sensory expression contour was similar. The on-vine group had slightly higher firmness, darker flesh color, lower water content, stronger flavor, and lighter overripe flavor scores for the entirety of the softening period.

### 3.6. Modeling Method to Analyze the Index Differences between Two Softening Methods

**Figure 10:**
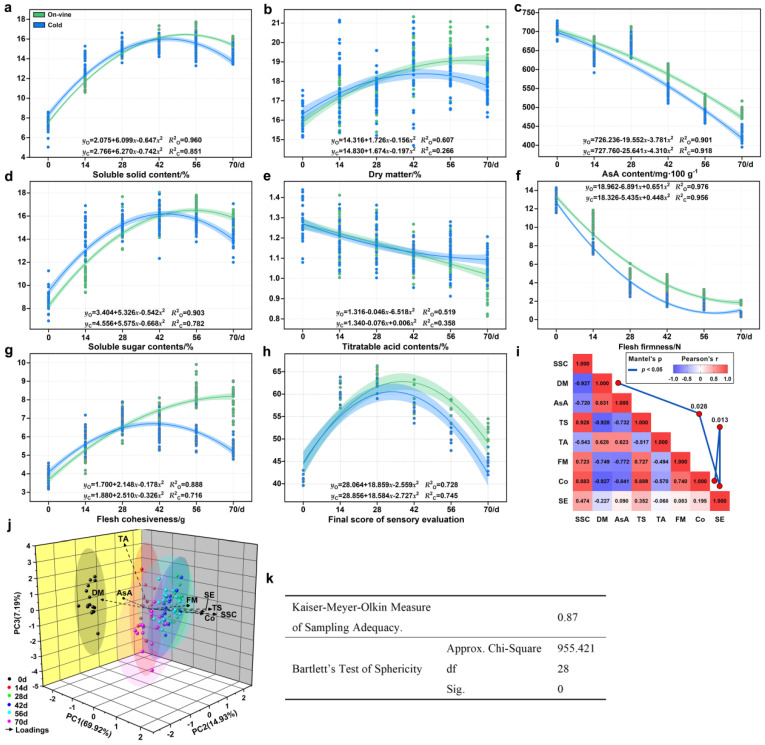
Mathematic and PCA modeling of test indices. (**a**–**h**) One-letter polynomial equation of regression of 8 test indices. (**i**) Correlation coefficient. (**j**) 3D load diagram of PCA. (**k**) Result of KMO and Bartlett’s Test.

In order to compare the differences between the two methods of soft ripening better over time, based on 8 test indices, the equation modeling of the two methods over time was carried out. By comparing the similarity of the two curves, it is speculated whether the test index will produce significant differences due to different treatments. Figure 10a–h shows the mathematical model of each developmental index in the soft ripening stage of ‘Ganlv-2’. We can observe that except for the titratable acid, the change trends of all other indices were almost the same, with only a certain difference in the degree of change and the time of the inflection point. The soluble solids, dry matter and flesh cohesiveness of the on-vine group overtook the cold group at 28 d, and the sensory evaluation was earlier, at 14 d. The soluble sugar content of the on-vine group exceeded the cold group at 48 d. Titratable acid showed opposite change trends with soluble sugars and soluble solids between the two groups, but the relative relationship shifted at 42 d. In general, the trends in most indices were similar, showing that the test indices do not show significant differences by treatment. The KMO measure of sampling adequacy of PCA was 0.87, which showed that the raw data were suitable for PCA. PCA showed that sensory evaluation was highly correlated with dry matter and flesh cohesiveness (0.028 and 0.013, *p* < 0.05), and that dry matter and flesh cohesiveness have a greater impact on sensory evaluation. Among all factors, the eigenvalue of two factors were >1 (5.594 and 1.194), contributing 69.92% and 14.93%, respectively. The cumulative contribution rate of the first three principal components reached 92.05%, representing the vast majority of variables (Figure 10i–k).

## 4. Discussion

Fruit quality is evaluated by the appearance and internal quality of fruit, which are the main factors by which the on-vine soft ripening of ‘Ganlv-2’ is determined. Appearance quality mainly refers to the weight and shape index of the individual fruit, and internal quality mainly includes dry matter, soluble solids, soluble sugar, titratable acid, and AsA contents [21,22]. Fruit softening is an extremely complex physiological process, which is influenced by starch degradation and changes of cell wall components and structures [47,48,49]. Firmness can reflect the compact degree and gap size of flesh cells to a certain extent, and cohesiveness can reflect the size of the bond between flesh cells to a certain extent. In general, the higher the cohesiveness, the better the texture and flavor, which is consistent with the results of this study [50]. The mechanical properties of the flesh are related to the cell structure, components, and state of inclusion (starch, cellulose, and pectin) [51]. In particular, cell wall pectin assumes a pivotal role in the structural properties of many fruit and vegetables, Huang et al. noted that higher pectin content could lead to greater firmness and cohesiveness of flesh, that the flesh cells were dense before fruit ripening, and that therefore the adsorption force between the sample and the probe is small [52]. The flesh becomes soft and juicy when the fruit softens. The adsorption force between the probe and the sample is increased during the return process, which causes more work to be done and higher cohesiveness. In this experiment, the firmness of ‘Ganlv-2’ decreased by 75.37% after 28 d in the cold group and reduced by 61.05% in the on-vine group. Multiple factors impact the softening rate of fruit. For instance, it is known that storage temperature and atmosphere (oxygen, carbon dioxide, and ethylene) can affect the softening rate in the main phase of rapid softening [53]. The change rate of flesh firmness was low before reaching the maturity stage, and the rate of flesh firmness in the soft maturity stage was sharply accelerated, which was similar to the research results of Burdon et al. on ‘Hayward’ [18]. At the early period of soft ripening, the fruit firmness decreased significantly, and then decreased slowly. However, it is different from similar studies on apple, where the delayed harvest of apples will cause faster softening than cold storage. We think that the difference in the ripening mechanism between climacteric and non climacteric fruit led to the discrepancy with our results [54,55]. The fruit in the on-vine group had a better ability to fight against fruit softening compared with those in the cold group, which improved the taste of fruit to a certain extent.

Kiwifruit is a type of climacteric fruit and soluble solid content is one of the main factors utilized for the judgment of fruit maturity. In this experiment, the trend of the change in the soluble solid content is similar to that of ‘Skinny Green’ kiwifruit. The variation trend of SSC before 44 d after harvest was a rapid increase in the early stage and a slow increase in the later stage of ‘Skinny Green’ kiwifruit, and it is similar to our result [56]. SSC generally showed an increasing trend, but we found that the fruit of the cold group and on-vine group decreased to a certain extent at 56 d and 70 d, respectively. We think this was mainly caused by the metabolic consumption of the fruit during soft ripening, and due to the degradation of cell wall components and the production of glucose by fruit senescence. At the same time, glucose and fructose produced by sucrose degradation enter multiple respiratory pathways to generate energy to maintain the life activities of fruit, so SSC will naturally decrease to a certain extent. The fruit in the on-vine group continued to absorb nutrients from the vine and hence there was a continual increase in the soluble solid content. There are different explanations for the impacts of on-vine soft ripening on the soluble solid content, and different findings of the same cultivar of kiwifruit have also been reported [57]. The titratable acid content can also reflect fruit maturity and be employed to identify storage quality [58]. Like the soluble solid content, the titratable acid content is also a critical component that determines taste and flavor. In this study, the titratable acid content of the on-vine group was slightly lower than that of the cold group, and this result was concurrent with the sensory evaluation [59,60].

AsA content is a crucial nutritional index of fruit and vegetable quality [61]. AsA loss during fruit ripening is also a physiological index of fruit senescence [62]. Furthermore, high acidity is beneficial for the maintenance of AsA [63]. In this experiment, the acidities were low in the fruit of both groups. Thus, the AsA content decreased as expected and exhibited a decreasing-increasing-decreasing trend in both groups, which was similar to *A. eriantha* ‘White’ [64]. In general, the content of AsA decreases continuously from maturity to soft ripening, but in this research, it is increased slightly in both groups at 28 d. We think that AsA as a kind of organic acid has an opposite change trend to soluble sugar during the soft ripening period. At 14 and 28 days, the increase rate of SSC in the two groups became slower at 14 d and 28 d, respectively, which may be related to the small increase of AsA content. The response time was different between two groups, which may be caused by different soft ripening methods, but the specific regulation mechanism is still unclear. There was no correlation of the dry matter, soluble solids, soluble sugar, and titratable acid with AsA contents in ‘Ganlv-2’ fruit during softening, but the soluble sugar content declined when the AsA content increased. Sugar is a key flavor substance that afflicts the postharvest edible quality of fruit [65]. The soluble sugar content reaches its peak when fruit begin to soften during the ripening process and decreases gradually as the fruit mature, which is similar to the results of Huang et al. [66]. This experiment elaborated that the on-vine soft ripening and the cold softening had similar functions of maintaining fruit quality.

**Figure 11:**
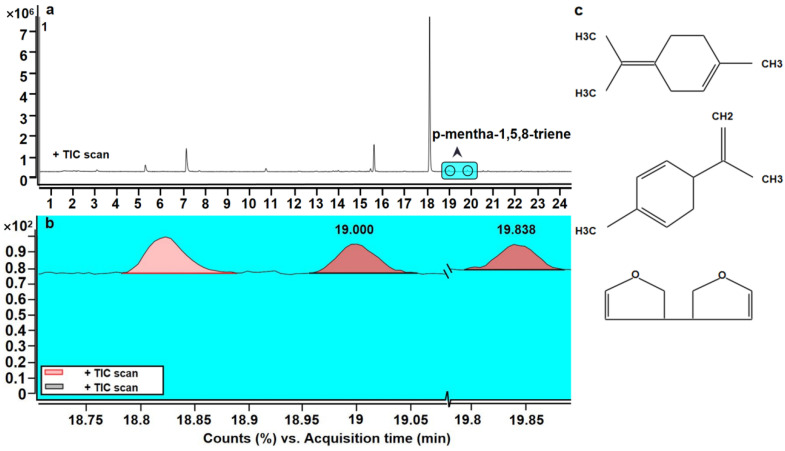
(**a**) Chromatogram of the on-vine group at 42 d. (**b**) Compound chromatography diagram. (overlaid) of p-mentha-1,5,8-triene (the peak at 19.000 and 19.838 min). (**c**) Molecular formulas of three isomers matched by NIST14 mass spectral database (p-mentha-1,5,8-triene, terpinolene and 3,3′-bifuran,2,2′,3,3′-tetrahydro- in proper order).

As fruit ripen, they change constantly during postharvest storage [21,22]. Currently, >80 aroma components have been reported in kiwifruit. The main components are methyl butyrate, ethyl butyrate, (E)/(Z)-hexenal, hexanal, (E)/(Z)-3-hexenol, and methyl benzoate. Prior researches have found that ethyl butyrate, 2-hexenal, and hexanal may be the three volatile components that determine the flavor of kiwifruit [67,68,69]. Ethyl butyrate was not detected but the other two species had a higher content in ‘Ganlv 2’ kiwifruit, which might be due to genetic differences. In this experiment, hexanal and 2-hexanal belonged to the category of C6 unsaturated aldehydes and alcohols, and both exhibited a typical grass flavor. They are mainly produced by linoleic acid and linolenic acid through the lipoxygenase pathway [47]. 2-hexanal was an aroma compound with the highest content detected at 0 d, and its content in the on-vine group decreased prominently with the extension of softening period. Meanwhile, 2-carene gradually became an aroma compound with the highest content, showing a typical aroma and thus reducing the grass flavor, which was consistent with the results of the sensory evaluation [70]. Nevertheless, 2-hexanal was always the most abundant aroma component in the cold group, which might be one of the reasons why the aroma score of the cold group was lower than that of the on-vine group. In the cold group, there were three kinds of aroma compounds (hexanethioic acid, s-methyl ester, and vinyl crotonate and terpinolene) on the 14 d, which did not appear in the remaining period of the cold group and the on-vine group. It was speculated that these two phenomena might be related to the stress response after cold stress, accompanied by a series of internal chemical reactions. Storing fruit at a low temperature delays the decrease in aldehyde content to some extent, which is similar to the results of this experiment [71], but the content of aldehydes reduced markedly and the content of terpenes enhanced dramatically in the on-vine group. It could be seen that different softening methods could affect the synthesis and variations of aroma compounds in ‘Ganlv-2’ kiwifruit. Additionally, with regard to fragrance quality, we found isomers of p-mentha-1,5,8-triene (C_10_H_14_), terpinolene (C_10_H_16_), and 3,3′-bifuran,2,2′,3,3′-tetrahydro- (C_8_H_10_O_2_). All of these aromatic substances appeared twice in the results of the total ion chromatography (the on-vine group at 42 d), and their peak areas were similar with all >80% similarity scores (Figure 11). A quantitative analysis was conducted on these substances using liquid chromatography. Moreover, the results were still similar to GC-MS. Therefore, it was not possible to determine whether individual aroma components in fruit are derived from specific isomers or whether several isomers exist simultaneously. The determination of the influence of these factors requires nuclear magnetic resonance, GC-olfactory technology, LC-mass spectrometry, and other techniques [72,73,74]. D-limonene is a monocyclic monoterpene, which is one of the ultimate products of mevalonic acid metabolism in plant cells. The content and variation of d-limonene in the two groups were similar, in line with the variation of soluble solids contents. It is hypothesized that there is a certain association between d-limonene and soluble solids contents, but the specific mechanism remains enigmatic.

The evaluation of food flavor is ultimately based on human senses as the main index. Food sensory evaluation technology collects the sensory data of a product from the evaluators and obtains the quantitative characteristics of the product through statistics and analysis [75]. Elortondo selected evaluators after testing the ability of participants to distinguish color, smell, and taste, and trained the evaluators for a measurement scale [76]. Pinto put forward a consistency test method based on Cronbach’s coefficient alpha on the premise of ensuring the quality of evaluators [77]. The results of the sensory evaluation and the fruit quality determination were comprehensively analyzed, showing that flesh firmness was positively correlated with flesh color, sensory firmness, and tissue state but negatively correlated with fruit juiciness, which was concordant with Zhang’s experimental results on ‘Hayward’ [78]. However, there were differences in flavor results, which might result from the two different softening methods, rather than the natural changes with the softening period and maturity. There are some human errors in sensory evaluation. Some scholars combine sensory evaluation with mathematical methods and instrumental analysis to reduce the error [79,80]. Sensory evaluation is a scientific evaluation of the sensory quality of a product, which can be utilized to guide the actual production. It is possible to organically combine social research methods with sensory evaluation. For example, the user survey method was adopted to determine the weight of sensory evaluation of samples by combining consumer acceptance with sensory evaluation [81,82]. Introducing the food sensory evaluation technology into social research methods is in line with the trend of discipline integration.

## 5. Conclusions

The on-vine soft ripening is convenient and efficient and can reduce transportation loss and storage costs. In this research, the on-vine soft ripening of ‘Ganlv-2’ was compared to that of traditional cold softening with fruit quality and aroma components as an index. The results revealed that the fruit quality produced from the two groups was similar, with the on-vine group having slightly better fruit quality than the cold group on some indicators. The sensory evaluation was basically similar, and the types of aroma substances in the on-vine group were more abundant, with 2-hexenal, hexanal, d-limonene and 2-carene as the dominant aroma compounds. The on-vine soft ripening breaks through the limitation that the existing cultivars need to be harvested and cooked before they can be eaten. This can facilitate the development of sightseeing agriculture and improve economic benefits. The results of this study have implications for both commercial operations and theoretical researchers. In conducting research, understanding the physiological state of the climacteric fruit at harvest is relevant to any findings on the soft ripening responses of the fruit. Commercially, temperature management, storage method and the response of fruit to them is a major component of the commercial handling of kiwifruit. This experiment provides a theoretical basis for the feasibility of the on-vine soft ripening of *A. eriantha*.

## Figures and Tables

**Table 1:** Sensory test scoring criteria.

Organoleptic Properties	Evaluation Criteria and Scores
Firmness	Soft ~ Tough (1–10)
Flesh color	Pale green ~ Blackish green (1–10)
Water content	Few ~ Rich (1–10)
Sweetness	Not sweet ~ Very sweet (1–10)
Sourness	Special sour ~ Not sour (1–10)
Kiwifruit flavor	Light ~ Special thick (1–10)
Acetic odor	Light ~ Special thick (1–10)
Overripe taste	Special thick ~ Little or none (1–10)

**Table 2:** The aroma substances of evident characteristics.

Aroma Substance	KI_CT_/KI_LT_	Aroma Description	Qualitative Method
Hexanal	1015/1040	Herbaceous flavor	MS/KI/St
Octanal	1243/1255	Orange flavor	MS/KI/St
D-Limonene	1008/Ne	Sweet fragrance, lemon flavor	MS/St
2-Carene	1023/Ne	Herbaceous flavor	MS/St

Note: ‘MS’ represents mass spectrum qualitative. ‘KI_CT_’ represents the calculated Kovats retention index. ‘KI_LT_’ represents the retention index of reference. ‘St’ represents the qualitative analysis of standard materials. ‘Ne’ indicates that no reference was found in the literature.

## Data Availability

The data presented in this study are available on request from the corresponding author.
